# Social Dancing and Incidence of Falls in Older Adults: A Cluster Randomised Controlled Trial

**DOI:** 10.1371/journal.pmed.1002112

**Published:** 2016-08-30

**Authors:** Dafna Merom, Erin Mathieu, Ester Cerin, Rachael L. Morton, Judy M. Simpson, Chris Rissel, Kaarin J. Anstey, Catherine Sherrington, Stephen R. Lord, Robert G. Cumming

**Affiliations:** 1 School of Science and Health, Western Sydney University, Penrith, Australia; 2 Sydney School of Public Health, University of Sydney, Sydney, Australia; 3 Institute for Health and Ageing, Australian Catholic University, Melbourne, Australia; 4 NHMRC Clinical Trials Centre, Sydney Medical School, University of Sydney, Camperdown, Australia; 5 Centre for Research on Aging, Health and Wellbeing, The Australian National University, Canberra, Australia; 6 Musculoskeletal Division, The George Institute for Global Health, Sydney Medical School, University of Sydney, Sydney, Australia; 7 Neuroscience Research Australia (NeuRA), University of New South Wales, Randwick, Australia; University of Cambridge, UNITED KINGDOM

## Abstract

**Background:**

The prevention of falls among older people is a major public health challenge. Exercises that challenge balance are recognized as an efficacious fall prevention strategy. Given that small-scale trials have indicated that diverse dance styles can improve balance and gait of older adults, two of the strongest risk factors for falls in older people, this study aimed to determine whether social dance is effective in i) reducing the number of falls and ii) improving physical and cognitive fall-related risk factors.

**Methods and Findings:**

A parallel two-arm cluster randomized controlled trial was undertaken in 23 self-care retirement villages (clusters) around Sydney, Australia. Eligible villages had to have an appropriate hall for dancing, house at least 60 residents, and not be currently offering dance as a village activity. Retirement villages were randomised using a computer generated randomisation method, constrained using minimisation. Eligible participants had to be a resident of the village, be able to walk at least 50 m, and agree to undergo physical and cognitive testing without cognitive impairment. Residents of intervention villages (12 clusters) were offered twice weekly one-hour social dancing classes (folk or ballroom dancing) over 12 mo (80 h in total). Programs were standardized across villages and were delivered by eight dance teachers. Participants in the control villages (11 clusters) were advised to continue with their regular activities. Main outcomes: falls during the 12 mo trial and Trail Making Tests. Secondary outcomes: The Physiological Performance Assessment (i.e., postural sway, proprioception, reaction time, leg strength) and the Short Physical Performance Battery; health-related physical and mental quality of life from the Short-Form 12 (SF-12) Survey. Data on falls were obtained from 522 of 530 (98%) randomised participants (mean age 78 y, 85% women) and 424 (80%) attended the 12-mo reassessment, which was lower among folk dance participants (71%) than ballroom dancing (82%) or control participants (82%, *p* = 0.04). Mean attendance at dance classes was 51%. During the period, 444 falls were recorded; there was no significant difference in fall rates between the control group (0.80 per person-year) and the dance group (1.03 per person-year). Using negative binomial regression with robust standard errors the adjusted Incidence Rate Ratio (IRR) was 1.19 (95% CI: 95% CI = 0.83, 1.71). In exploratory post hoc subgroup analysis, the rate of falls was higher among dance participants with a history of multiple falls (IRR = 2.02, 95% CI: 1.15, 3.54, *p* = 0.23 for interaction) and with the folk dance intervention (IRR = 1.68, 95% CI: 1.03, 2.73). There were no significant between-group differences in executive function test (TMT-B = 2.8 s, 95% CI: −6.2, 11.8). Intention to treat (ITT) analysis revealed no between-group differences at 12-mo follow-up in the secondary outcome measures, with the exception of postural sway, favouring the control group. Exploratory post hoc analysis by study completers and style indicated that ballroom dancing participants apparently improved their gait speed by 0.07 m/s relative to control participants (95% CI: 0.00, 0.14, *p* = 0.05). Study limitations included allocation to style based on logistical considerations rather than at random; insufficient power to detect differential impacts of different dance styles and smaller overall effects; variation of measurement conditions across villages; and no assessment of more complex balance tasks, which may be more sensitive to changes brought about by dancing.

**Conclusions:**

Social dancing did not prevent falls or their associated risk factors among these retirement villages' residents. Modified dance programmes that contain "training elements" to better approximate structured exercise programs, targeted at low and high-risk participants, warrant investigation.

**Trial Registration:**

The Australian New Zealand Clinical Trials Registry ACTRN12612000889853

## Abstract 2

In a cluster-randomized trial, Dafna Merom and colleagues have studied the potential for provision of social dancing classes to reduce the incidence of falls in elderly people.

## Author Summary

**Why Was This Study Done?:**

- Activities that challenge balance are recognized as efficacious fall prevention strategies.
- Pilot studies have demonstrated that a variety of dance styles can improve balance and gait speed of older adults, but no studies have examined whether dance interventions can reduce the number of falls.

**What Did the Researchers Do and Find?:**

- We invited all adults who lived independently in 23 retirement villages across Sydney, Australia, to participate in social dance classes (folk dance or ballroom) twice weekly over 12 mo, in total 80 h; 12.3% of the residents expressed interest in the program.
- We randomly assigned the retirement villages to receive the social dancing, (12 villages, 279 participants) or to a “wait-listed” comparison group (11 villages, 251 participants).
- We found that social dancing was not effective in reducing the number of falls; nor did it improve a variety of fall-related risk factors (e.g., balance, lower leg strength, cognitive processing speed, or task shifting), apart from a small apparent improvement in gait speed, particularly among ballroom participants.
- We also found that older adults who had multiple falls in the year prior to the study and received the dance program seemed to fall more often than their counterparts in the comparison group.

**What Do These Findings Mean?:**

- This large-scale pragmatic trial suggests that social dance, as delivered in this trial, should not be considered as a fall prevention strategy.
- The mixture of participants with a variety of fall-risk levels, the inclusion of active participants, and the relatively low attendance, on average 51% of the classes prescribed, may explain these results.
- We suggest that a modified social dance program that contains “training elements” of structured exercise, particularly balance, targeting low and high risk groups separately, should be tested to ascertain whether dance is an effective fall prevention strategy.

## Introduction

Falls are one of the most common age-related health problems for older adults and a common cause of injury-related hospitalisation, loss of independence, and poor quality of life [1,2]. It is now well recognised that exercise as a single intervention can help to address the physiological deficits that are part of the multifactorial etiology of falls and can reduce fall rates by approximately 30%, particularly with a focus on balance challenging exercises [3–5].

Participation in specific fall prevention exercise, however, is still low; for example, in 2009 in New South Wales (NSW), Australia, only six percent of adults aged 65 y and over undertook specific balance exercises in the past week, and only 12% reported some form of strength training [6]. However, the evidence-based principles of balance or strength training may be present within a single activity type, such as Tai Chi, which can reduce fall rates by 37% [7]. Tai Chi integrates multiple physical and cognitive elements that are claimed to be “synergetic” rather than a composite of separate components [8]. Dance also shares this “holistic” practice approach. Dance is a complex sensorimotor rhythmic activity integrating multiple physical, cognitive, and social elements, all of which have the potential to ameliorate a wide range of physical and cognitive fall risk factors. For many people, dance is also an engaging social activity and thus has advantages over many simple repetitive strength and balance regimens that are often undertaken alone.

It has been suggested that dance may be an effective fall prevention strategy; a claim primarily inferred from studies of exceptional balance abilities of professional young dancers [9]. Since then, several dance-based studies involving older adults have been published [10], supporting the benefits of dance in improving gait and balance—two of the strongest risk factors for falls in older people [11]. For example, cross-sectional studies have shown that seniors who dance have superior balance and gait characteristics compared to aged-matched controls [12,13]. A recently published observational study among 1,683 community-dwelling older Japanese people [14] showed that regular dancing for at least one year was associated with a 70% lower history of one or more falls, but the observational nature of this study and the retrospective assessment of falls provided limited evidence for causal association. Small-scale randomised controlled trials (RCTs) of ≤50 participants have also shown that a variety of dance styles resulted in improved balance and gait speed in older people [15–19].

These study findings and quasiexperimental trials [20–23] are encouraging, but no studies to date have examined whether dance interventions can reduce the incidence of falls. The evidence falls well short, therefore, of that required for large-scale promotion of dance as a public health fall prevention initiative. This study primarily aimed to determine whether community folk or ballroom dancing is effective in: i) reducing the number of falls in retirement village residents, and ii) improving Trail Making cognitive functions tests, thereby elucidating possible mechanisms for this putative fall prevention strategy in this group. Additionally, we wished to assess the impact upon physical function and physical and mental health-related quality of life of such an intervention [24]. To address a major barrier to exercise participation in this age group, lack of transport [25], the trial was implemented in retirement villages to facilitate easy access to dance venues.

## Methods

The Western Sydney University Human Research Ethics Committee (ref: 9468) approved this research project. A parallel group, cluster RCT was undertaken in self-care retirement villages (clusters) around Sydney, Australia, between October 2012 and November 2014. The trial was registered prior to commencement with the Australian New Zealand Clinical Trials Registry (ACTRN12612000889853).

### Recruitment and Participants

Invitation letters were sent to 112 self-care retirement villages within the postcodes of the Sydney metropolitan area that are listed by the NSW Retirement Village Association (250 villages in total). Eligible villages had to have an appropriate hall for dancing (i.e., safe floor, appropriate size), house at least 60 residents to afford a minimum group size of 12 participants, and not be currently offering dance as a village activity. The project was advertised by posters placed in key village locations. An information session was held at each village during which participants were introduced to the study rationale, the types of social dancing (a video) that would be offered in the intervention, and the anticipated dates and times the dance classes would be held. Interested participants left their telephone contact details with the research staff to determine eligibility and to schedule baseline assessments.

Eligible participants had to be a resident of the village; be able to walk at least 50 m; agree to undergo physical and cognitive testing; plan to stay in the village for the next 12 mo; and obtain medical clearance to participate in the study. Participants were excluded if they planned to leave the village for three months or more during the trial period, or if they scored <24 on the Mini Mental State Examination (MMSE) in the baseline assessment indicating cognitive impairment [26].

### Intervention

Dance classes were offered for one hour, twice a week, for a total of 80 h over 12 mo (allowing for short breaks). We assumed that all dance styles would be equally effective because they share similar principles: movements are synchronized to music and organized into spatial patterns, which tend to be modular in organization (i.e., composed of discrete sections that are repetitive). Participants in the 12 intervention villages were offered one of two major social dancing styles: Folk dancing (five villages), which included dances from the United Kingdom, United States, France, Italy, Israel, and Greece; or ballroom dancing (seven villages), which included dances such as Rock and Roll, Foxtrot, Waltz, Salsa, and Rumba. The allocation to style was based on logistical considerations (e.g., teacher of any style that was able to fit in with the village’s time-table and lived within a short distance of the village). The aim was to test the efficacy of dance programs commonly available in the community. Four folk dance teachers and four ballroom teachers delivered the program which was standardised via two workshops, a guidebook, and a DVD developed by the dance coordinators. Over the 12 mo of the dance intervention, cognitive complexity and cardiovascular effort were gradually increased. Teachers were asked to record attendance at every class on a pre-prepared teachers’ diary given at the start of the program. They were asked to contact participants if they did not show up for more than four classes (two wk) in a row to verify the reason and to record it in the diary. Any adverse event incidence during the class had to be recorded in the booklet.

### The Control Group

Participants in the 11 control villages were advised to continue with their regular activities, and asked not to join a dance class during the trial period. Controls were placed on a wait list for the dance classes at the end of 12 mo. Physical activity was measured at baseline, six, and 12 mo to monitor changes over the trial period in both groups using the validated Incidental and Planned Exercise Questionnaire (IPEQ) for older adults [27].

To keep participants engaged, all participants (i.e., control and intervention villages) received a monthly newsletter (12 in total) in which we introduced the study investigators and research team (one in each month), provided an update on recruitment from the study field manager, a health message (not related to exercise), the study contact details, and a reminder to complete the next month’s falls calendar.

### Randomisation and Blinding

Retirement villages were randomised by the trial statistician using a computer generated randomisation method, constrained using minimisation [28]. That is, each village was allocated with a probability of 0.8 to the group that minimised the imbalance of the means of two baseline tests, Physiological Performance Assessment (PPA) z-scores and Trail Making Test Part B (TMT-B) time, between intervention and control groups, using two strata (below and above the anticipated median) for each variable. The trial statistician (JMS) advised the study coordinator (EM) of the village’s allocation, and the study coordinator arranged the delivery of the intervention. Allocation was thus concealed from the research team that were recruiting villages and participants and performing the baseline assessments. Allocation to dance style was arranged by the study coordinator based on teacher availability and the teachers’ proximity to each village.

During the trial, participants were asked not to reveal details about the program to research staff. The recording of falls from participant diaries was performed by research staff blind to allocation. However, research staff administering the 12-mo assessment were not blinded. Statistical analysis of falls, Trail Making Tests (TMTs) and quality of life were undertaken blind to allocation.

### Primary Outcome Measures

#### Number of falls during the 12 mo trial

A fall was defined as “unintentionally coming to rest on the ground, floor, or other lower level” [29]. Participants were asked to record “F” (fall) or “N” (no fall) each day using monthly calendars (diaries), which were returned by mail at the end of each month. Participants who reported a fall were interviewed by telephone to obtain details about where the fall(s) occurred; whether the fall resulted in injuries; and whether any treatment was sought. Participants who did not return their calendars within two wk were telephoned by study researchers and verbal responses were recorded. At the end of the call, they were also requested to return their calendar by mail to maintain completeness.

#### Time to complete TMTs Parts A and B [30]

The TMT Part A measures processing speed and involves participants connecting consecutive numbers (e.g., 1-2-3). Part B provides a measure of executive function or “task shifting,” and involves participants connecting alternate letters and numbers (e.g., 1-A-2-B). The difference in time between the two parts (B minus A) was calculated to isolate the executive component of this test.

### Secondary Outcome Measures

The PPA [31] is a validated measure of physiological fall risk. PPA scores are computed from weighted performance scores from five tests: vision (edge contrast sensitivity), peripheral sensation proprioception, lower extremity strength (knee extension), simple reaction time using a figure press as the response, and standing balance measured by body sway when standing on a medium-density foam rubber mat with eyes open.

Functional mobility was assessed with the Short Physical Performance Battery (SPPB) [32] which includes tests of side-by-side, semitandem, and tandem standing for at least 10 s, walking speed over 3 m, and time to complete five chair rises.

Gait speed (m/s) was calculated from the fastest time to walk 3 m from three trials.

Health-related quality of life was measured at baseline, three and 12 mo using the self- reported SF-12 survey V2 [33]. Mean scores for each of the eight domains (physical functioning, role physical, bodily pain, general health, vitality, social functioning, role emotional, and mental health) were combined into two summary health scores; the Physical Component Summary (PCS) and the Mental Component Summary (MCS). These scores were normalised to the general population by linear transformation, (mean 50, standard deviation [SD] 10) according to the scoring manual [34], and reported by trial group. Higher scores indicate better health-related quality of life. A difference in PCS or MCS of ≥5 (that is, 0.5 SD) is considered to be clinically meaningful [35].

### Sample Size

We hypothesized that dance would lead to a 37% reduction in incidence of falls, as this was the pooled estimated effect of Tai Chi on fall rates in the 2009 version of the relevant Cochrane review (the current version at the time of trial design) [7]. A sample size of 518 (259 per group) was required in order to have 80% power to detect the above 37% fall rate reduction at the two-tailed 5% significance level. This assumed an incidence of 0.85 falls per person per year in the control group, a dispersion parameter of 0.79, an intracluster correlation coefficient of 0.015, 90% completion of falls diaries, and an average cluster size of 22 participants, which is achievable by recruiting 23–24 retirement villages. Our initial calculation was made using a cluster size of 12 participants; a sample of 229 per arm or 458 in total was required for this scenario with all other parameters remaining as above, but required recruitment of 38 retirement villages, which was not manageable.

### Statistical Analysis

Baseline characteristics of the intervention and control groups were summarised. We used negative binomial regression to estimate the effect of the intervention on the number of falls in 12 mo, using Generalized Estimating Equations (GEE) to allow for clustering. For adjusted primary analyses, we also allowed for age, sex, educational attainment, MMSE score, PPA score, and dancing status.

We undertook post hoc analyses to test whether there was a differential effect of the intervention by baseline history of falls and dance style. We tested differences between the groups at 12 mo for TMT tests, PPA total scores, and PPA components presumed to be influenced by the intervention (i.e., reaction time, proprioception, leg strength, and postural sway), the SPPB total score, repeated timed sit to stand, gait speed, and health-related quality of life using GEEs to allow for clustering and adjusting for baseline scores and the covariates listed above.

Primary analyses were conducted on an ITT basis. Missing data were estimated by multiple imputation using chained equations (with 30 imputed datasets) [36]. Missing quality of life items within domains were imputed with mean population weights [37], and sensitivity analyses were undertaken using the three-month observation carried forward and for complete cases. Adherence to dance programs was calculated by dividing the number of sessions each participant attended by the total number of sessions offered. Additional sensitivity analyses were undertaken for those who attended 12-mo measurements (“completers”), by adherence and dance style. We used Stata version 10 and SAS software version 9.3.

### Participant Involvement

As part of the intervention design, we trialled the program among nine community-dwelling older adults for 12 wk. These individuals provided feedback on the dance repertoire, music selection, method of instruction, and other issues (e.g., degree of physical comfort while practising certain movements). In addition, as part of the instructor hiring process, 20 long-term senior folk dancers volunteered their time to participate in and provide feedback on class demonstrations undertaken by potential instructors. A representative of the group was asked to collate feedback after each class and to communicate their impressions to the selection panel. During the trial, teachers asked participants to rate their perceived exertion at class number 20, 40, 60, and 79. This information was used to determine the progressiveness of the program in terms of aerobic effort. At the end of the study, participants received a personalised letter communicating the changes in “fall risk” status based on their PPA scores at baseline and follow-up in comparison to population age-appropriate norms. Lastly, all participants were invited to take part in focus groups about the program at study end. The results of focus groups are currently being processed.

## Results

**Fig 1:**
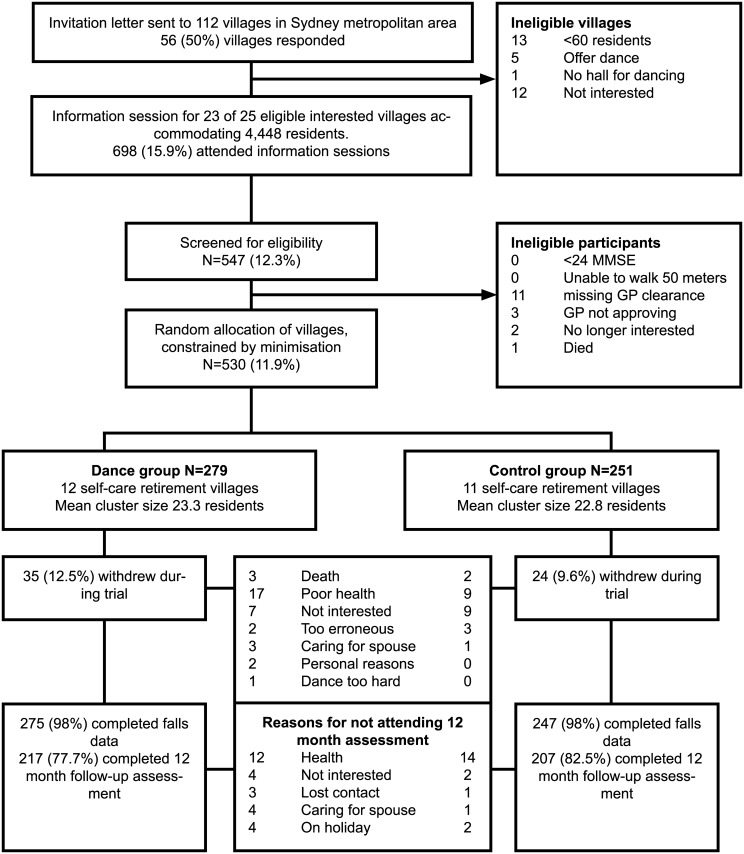
Cluster allocation and participant flow by study arm. *MMSE indicates Mini-Mental State Examination.

Fig 1 presents the flow of participants over the trial period. Of the 112 villages, 56 (50%) responded to our invitation; 12 (10%) declined, and 19 (17%) were ineligible, leaving 25 potential villages. Two villages underwent changes in management and were no longer interested, leaving 23 villages. Given that the desired sample size of 518 participants had been achieved, we ceased recruiting.

### Retention and Attendance

Of the 530 participants randomised, 522 (98%) provided data on falls and 424 (80%) attended the 12-mo assessment (Fig 1). The most common reasons for attrition (Fig 1) were poor health (11.3%), a loss of interest in the trial (4.5%), loss of contact or on holiday (1.9%), and caring for a spouse/family member (1.5%). There were no significant differences between the groups by reasons of attrition. Only one participant withdrew due to a difficulty in performing the dances. There were no adverse events associated with participation in the dance programs.

The average cluster size at baseline was 23 participants (Table 1) and ranged from 13 to 43 participants. Retention to the 12-mo assessment varied markedly by village ranging from 60% to 92% (Table 1). The median attendance to sessions was 56%, (IQR 26–77%) or approximately 45 sessions. The median attendance was somewhat lower for folk (55%) than ballroom dancing (60%). Overall, study-end retention was somewhat higher in the control group (82%) than in the dance group (78%). When stratified by dance style, retention was significantly lower among folk dance (71%, 95% CI: 61, 79) than ballroom dance participants (82%, 95% CI: 76, 87) or controls (82%, 95% CI: 78, 87, χ^2^_(2)_ = 6.5, *p* = 0.04).

**Table 1:** Intervention (dance) and wait-listed (control) villages (clusters) by cluster size baseline and follow-up number of participants and adherence to dance sessions.

	Dance villages				Control villages			
Village No.	Pop. ^a^, *n*	Baseline cluster size *n* (%)	Study-end retention *n* (%)	Attendance ^b^, %	Village No.	Pop.^a^, *n*	Baseline cluster size *n* (%)	Study-end retention, *n* (%)
3 ^c^	85	21 (25)	15 (71)	53	1	110	14 (13)	13 (93)
6 ^c^	114	14 (12)	9 (64)	57	2	96	19 (20)	12 (63)
7	350	43 (12)	35 (81)	51	4	240	27 (11)	19 (70)
8	140	18 (13)	16 (89)	49	5	300	33 (11)	29 (88)
9 ^c^	250	22 (9)	18 (82)	49	11	127	22 (17)	20 (92)
10 ^c^	120	13 (11)	10 (77)	59	12	350	26 (7)	18 (69)
14	133	25 (19)	16 (64)	54	13	113	38 (34)	36 (95)
15 ^c^	276	25 (9)	15 (60)	38	16	300	22 (7)	18 (82)
17	164	26 (16)	20 (77)	48	18	180	13 (7)	11 (77)
20	300	32 (11)	28 (88)	55	19	175	20 (11)	17 (85)
21	210	25 (12)	23 (92)	62	22	150	17 (11)	14 (86)
23	135	15 (11)	12 (80)	61	-	-	-	-
Total	2,277	279 (12)	217 (78)	51^d^		2,141	251 (12)	207 (82)

^a^ = Population size

^b^ = attendance was calculated as proportion of classes attended from total number of classes delivered, between 79 to 80 classes depending on the village

^c^ = village received folk dancing

^d^ = mean attendance

Compared with control participants, those in the dance group were older, had a lower MMSE score, were more likely to be participating in dance, and less likely to be highly active (Table 2). Health-related fall risk factors were balanced between the groups, with the exception of postural sway and falls risk, with more participants in the dance group classified as being at moderate to high risk according to population normative data (Table 2).

**Table 2:** Characteristics of study participants at baseline by group.

	Dance		Control		Total Sample	
	*N* = 279		*N* = 251		*N* = 530	
	*n* (%)		*n* (%)		*n* (%)	
**Demographics**						
Age >80 years	119	(43)	89	(35)	208	(39)
Female sex	231	(83)	217	(86)	448	(85)
**Country of Birth**						
Australia	193	(69)	193)	(77)	386	(73)
English-speaking	52	(19)	40	(16)	92	(17)
Non-English speaking	34	(12)	18	(7)	52	(10)
**Highest level of educational attainment**						
Year 10 or below	88	(31)	103	(41)	191	(36)
Completed High school/Technical And Future Education (TAFE) ^a^	146	(52)	127	(51)	273	(51)
University degree	45	(16)	21)	(8)	66	(12)
**Living alone**						
Yes	175	(63)	142	(57)	317	(60)
**Falls in past year**						
0 falls	202	(73)	180	(72)	382	(72)
1 fall	48	(17)	45	(18)	93	(18)
≥2 falls (“multiple fallers”)	27	(10)	26	(10)	53	(10)
**Number of chronic conditions**						
≥2 chronic conditions	190	(68)	180	(72)	377	(71)
**Diseases known to increase falls**						
Stroke	28	(10)	16	(6)	44	(8)
Parkinson Disease	3	(1)	2	(1)	5	(1)
Arthritis	154	(55)	151	(60)	305	(58)
Diabetes	34	(12)	24	(10)	58	(11)
Depression symptoms (GDS ^b^ ≥5)	43	(15)	35	(14)	78	(15)
**Poor vision** (Edge contrast sensitivity ≤16) ^c^	12	(4)	5	(2)	17	(3)
**Medication use**						
>5 medications	117	(42)	95	(38)	212	(40)
Psychoactive medications	17	(6)	12	(5)	29	(5)
**PPA risk profile** ^d^						
No risk	80	(29)	83	(33)	163	(31)
Low	88	(31)	104	(41)	192	(36)
Mild	64	(23)	43	(17)	107	(20)
Moderate to High	47	(17)	21	(8)	68	(13)
**Cognitive status (MMSE)** ^e^						
28–30	183	(65)	188	(75)	371	(70)
<27	96	(34)	63	(25)	159	(30)
**Exercise level**						
≥3.0 h/wk of planned exercise	164	(60)	150	(60)	314	(59)
≥14.0 h/wk of physical activity	230	(83)	211	(84)	441	(83)
**Dancing status**						
Currently dancing	23	(8)	9	(4)	32	(6)
Never danced	72	(26)	74	(29)	146	(28)

^a^ = TAFE is a vocational college in Australia

^b^ = GDS = Geriatric Depression Score

^c^ = Edge contrast sensitivity is part of the Physiological Performance Assessments (PPA) tests

^d^ = Physiological Performance Assessment risk profile was calculated from participants’ PPA z-score compared to age-specific population norms

^e^ = MMSE = Mini Mental State Examination

Within the intervention group, lower proportions of those living in villages that received folk rather than ballroom dancing were aged 80 y or above and born in Australia. We note that higher proportions of those in villages that received folk rather than ballroom dancing were over 80-y-old, living alone, had a medical history of stroke or arthritis, had more than two chronic conditions, and worse physiological fall risk profile, which are all known risk factors for falls (S1 Table).

### Change in Physical Activity during the Trial

At 6 mo, participants in the control group (*n* = 232) had increased their time spent in weekly planned exercise (not including walking), on average, by 18 min (95% CI: 4, 40); did not change significantly the time spent undertaking planned walks (−12 min, 95% CI: −30, +4); and appeared to have increased incidental physical activity (i.e., walking for errands, outdoor and indoor chores) on average by 113 min (95% CI: −2, 234). Those in the intervention group (*n* = 247) had increased planned exercise, on average, by 110 min (95% CI: 90, 138), as expected; did not change significantly the time spent undertaking planned walks (+5 min, 95% CI: −10, +20); and increased incidental physical activity by 142 min (95% CI: 34,249). These changes remained similar at 12 mo, although in both groups those remaining in the study further increased their incidental physical activity significantly, on average, by 134 min (95% CI: 57, 210), with the exception of those receiving folk dancing where incidental physical activity apparently declined from 6 to 12 mo (−42 min, 95% CI: −27, 150).

### The Effect of Social Dancing on Falls

The mean follow-up time for falls for the 522 participants with some fall data was 47.9 wk (SD = 11.5), shorter in the dance group (46.9, SD = 12.9) than in the control group (49.0, SD = 9.8, t_520_ = 2.12, *p* = 0.03). During this period, 444 falls were recorded, and the unadjusted incidence rate was 0.85 falls per person-year. A total of 297 (57%) participants experienced no falls, 143 (27%) experienced one fall, and 82 (16%) experienced two or more falls.

Table 3 shows the distribution of falls by group allocation. The fall rate was lower (0.80 per person-year) in the control group than in the dance group (1.03 per person-year), giving an unadjusted IRR for the dance group of 1.34 (95% CI: 0.98, 1.83) allowing for clustering. After also adjusting for age, sex, educational attainment, baseline MMSE, PPA falls risk score, and current dancing at baseline, the IRR was 1.19 (95% CI: 0.83, 1.71).

**Table 3:** Number of falls and incidence of falling among study groups and by baseline falls history.

	Dance (*N* = 275)	Control (*N* = 247)	Unadjusted	Adjusted
	Falls (rate ^a^)	Falls (rate)	IRR ^b^ (95% CI)	IRR ^c^ (95% CI)
All study participants (*n* = 522)	257 (1.03)	187 (0.80)	1.34 (0.98–1.83)	1.19 (0.83–1.71)
Falls in the past 12 mo				
No fall (*n* = 377)	142 (0.78)	103 (0.61)	1.35 (0.83–2.21)	1.19 (0.67–2.10)
1 fall (*n* = 92)	34 (0.81)	44 (1.06)	0.77 (0.42–1.38)	0.78 (0.45–1.35)
≥2 falls (*n* = 51)	74 (3.12)	40 (1.78)	1.69 (1.17–2.44)	2.02 (1.15–3.54)

^a^ = fall rates per person-year

^b^ = IRR for dance group compared to control group, allowing for cluster

^c^ = Adjusted for age, sex, educational attainment, baseline MMSE, dancing status at baseline, fall risk at baseline.

The adjusted model included 521 participants due to one person with missing data on dancing status, and the adjusted model comparing previous fallers included 141 participants due to two participants with no data on history of falls.

Exploratory post hoc subgroup analysis revealed there was no significant effect of social dancing in those who did or did not report falling in the 12 mo preceding the baseline assessment. However, in those who fell multiple times in the year before the trial, those randomised to dancing fell significantly more than the controls during the trial period (adjusted IRR = 2.02, 95% CI: 1.15, 3.54, *p* = 0.23 for interaction). Post hoc subgroup analysis also showed a significantly higher rate of falls in the folkdance intervention group than in controls (adjusted IRR = 1.68, 95% CI: 1.03, 2.73), but no difference in fall rate between ballroom dancing and control participants (adjusted IRR = 0.92, 95% CI: 0.65, 1.29) (S2 Table). Dance participants whose attendance was high (*n* = 142, ≥45 sessions) had the lowest incidence of falls (0.73 per person-year, (S2 Table), particularly among ballroom participants (S2 Table) who had the lowest incidence (0.66 falls per person-year, adjusted IRR = 0.71, 95% CI: 0.48, 1.05). In comparison, the fall rate in folk dancers with high attendance was not different to the control group (0.85 person-year, adjusted IRR = 1.14, 95% CI: 0.75, 1.74).

### The Effect of Dance on TMTs and Secondary Outcomes

There were no intervention effects for the TMTs in the ITT analysis (Table 4). The adjusted between-group difference in TMT-B was 2.8 s (95% CI: −6.2, 11.8). No between-group differences in TMTs were found among study completers (S3 Table).

**Table 4:** Difference between baseline and “study-end” scores for the TMT and secondary outcomes by study allocation (*n* = 522).

	Dance		Control		Intervention effect: adjusted ^a^ between-group difference at 12 mo (95% CI)		
	Baseline Mean (SD)	12 mo Mean (SD)	Baseline Mean (SD)	12 mo Mean (SD)			*P* ^a^
TMT ^b^ A (s)	44.8 (18.3)	47.5 (22.9)	40.0 (14.5)	42.0 (18.6)	0.8	(0.6, 1.0)	0.86
TMT ^b^ B (s)	125.3 (66.9)	130.4 (75.7)	120.9 (69.8)	119.9 (65.6)	2.8	(−6.2, 11.8)	0.54
TMT ^b^ difference (s)	80.4 (56.1)	82.9 (65.3)	80.8 (63.0)	77.9 (56.5)	1.0	(−8.5, 10.5)	0.84
PPA ^c^ score	0.77 (1.29)	1.02 (1.43)	0.49 (1.07)	0.69 (1.23)	0.16	(−0.15, 0.46)	0.31
Proprioception (degrees)	1.91 (1.29)	2.37 (1.81)	1.81 (1.15)	2.21 (1.54)	0.15	(−0.20, 0.49)	0.41
Leg strength (kg)	22.1 (12.7)	25.2 (12.1)	22.9 (10.8)	25.8 (12.3)	0.3	(−4.0, 4.5)	0.91
Postural sway (mm)	172 (145)	175 (191)	138 (106)	129 (100)	1.24	(1.00, 1.54) ^f^	0.05
Reaction time (s)	257 (67)	274 (95)	253 (54)	267 (73)	1.00	(0.95, 1.05) ^f^	0.81
SPPB ^d^ score	10.2 (1.8)	7.9 (4.8)	10.6 (1.6)	8.8 (4.3)	−0.6	(−1.51, 0.33)	0.21
Repeated sit-to-stand	12.7 (4.5)	17.8 (10.8)	12.3 (4.3)	16.1 (9.9)	1.5	(−0.7, 3.8)	0.19
Gait speed	0.94 (0.25)	0.90 (0.28)	1.01 (0.22)	0.91 (0.24)	0.02	(−0.05, 0.11)	0.68
Quality of life ^e^							
Physical component score	43.0 (8.8)	39.8 (10.9)	44.3 (8.7)	40.8 (10.8)	0.0	(1.8, 1.9)	0.96
Mental component score	52.1 (8.4)	49.4 (10.8)	51.9 (7.6)	50.3 (9.5)	−0.9	(−2.9, 2.0)	0.34

^a^ = Between-group difference in 12 mo scores for all outcomes, except for “one leg stance”, were assessed using GEE adjusted for age, sex, educational attainment, and baseline outcome values, MMSE score and dancing status, and accounting for retirement village clustering effect. Between-group differences in “one leg stance” were estimated using Tobit regression models with the same covariates as above, robust standard errors accounting for retirement village clustering effect, and accounting for censored values (all participants given a maximum value of 10 s even if they could stand longer).

^b^ = Trail Making Test

^c^ = Physiological Performance Assessment

^d^ = Short Physical Performance Battery

^e^ = SF-12 v2 survey

^f^ = represents antilogarithm of regression coefficient as GEE using logarithmic link function; value represents proportional difference in outcome between the dance and control groups.

Further, ITT analysis revealed no between group differences at 12-mo follow-up in the secondary outcome measures, with the exception of postural sway (Table 4). Mean sway in the control group declined by 9 mm, indicating improvement, whereas in the dance group the mean sway increased by 3 mm, resulting in apparently significant between-group differences (12 mm) in favour of the controls (*p* = 0.05).

The exploratory analysis among study completers (*n* = 424) showed a larger between-group difference in postural sway (S3 Table). Mean gait speed tended to improve in the dance group (+0.03 m/s) and decline in the controls (−0.3 m/s), but this difference was not statistically significant. Post hoc analysis of changes from baseline to study-end stratified by dance style (S4 Table) revealed that, compared to the control group, folk dance participants performed significantly worse on the SPPB test and five chair rises. On the other hand, ballroom dancing participants seemed to improve their gait speed by 0.07 m/s, significantly more than control participants whose mean gait speed declined (*p* = 0.05).

### Quality of Life

Baseline physical health (PCS) in the study cohort was substantially lower than the norm of 50 for the general population, but baseline mental health (MCS) was consistently higher (Table 4). Quality of life scores decreased for both groups between baseline and study end (Dancing group PCS −3.2, MCS −2.7; Control group PCS −3.5, MCS −1.6), and the difference was not statistically significant; (PCS *p* = 0.97; MCS *p* = 0.36). No significant differences were observed in sensitivity analyses of SF-12 scores for completers (S3 and S4 Tables), or when using the last observation carried forward. Stratification by dance style (S4 Table) revealed a non-significant decline in mental health score only among the folk dancing participants (−2.1 points, *p* = 0.18). Cost-effectiveness analyses will not be performed in view of the lack of benefit observed.

## Discussion

In this study, participation in social dancing programs did not reduce falls in older adults living independently in retirement villages. Furthermore, the intervention did not lead to significant improvements in cognitive risk factors as measured with the TMT-B, or physiological fall risk as measured by the PPA. The only improvement among intervention participants was a small apparent increase in gait speed, particularly among the ballroom dancing group, but worse performance than control on the postural sway test. Post hoc analysis revealed that multiple fallers, i.e., in the year prior to randomisation, in the social dancing group had a significantly higher rate of falls than multiple fallers in the control group. There were no significant differences between the groups in physical or mental health-related quality of life.

### Main Results in the Context of Other Research

A possible explanation for the lack of an intervention effect in this study is that social dancing does not contain the necessary “training elements” to achieve a sufficient balance challenge needed for reducing falls [38]. Previous RCTs that showed improved balance through dance were designed as “dance-training” [15,17,20]. For example, the RCT by Federici and colleagues [15] included specific dynamic and static balance exercises and coordination exercise along with Caribbean dancing [15]; in contrast, our program had no additions of balance exercises and the ballroom dances or folk dances do not provide opportunities for standing without support or standing on one leg for a long time, which are elements identified as high balance challenge exercises [3,38]. The dance programs, however, provided many elements of stepping practice [38], a factor that may have induced the improvement in gait speed. The improvement in gait speed may be important, as gait speed is a strong predictor not only for falls [11] but also for survival [39,40]. However, the small improvements in gait appear to have been offset by the reduced performances in the balance measures in the context of falls prevention.

A second possible reason for the lack of an intervention effect is an inadequate training dose, and that in order for social dancing to be effective, a higher dose over a longer period may be needed to be efficacious. Dose of exercise has been identified as a critical component of successful fall prevention programs, with a cut-point of about 56 h over the trial period differentiating between efficacious and nonefficacious programs [3]. In our study, only 39% of the dance participants achieved this threshold, and those with high adherence had a lower incidence of falls, particularly in the ballroom dancing group. However, lower fall risk in the high-attendance participants may explain this finding.

A third reason for the null finding could be that participants in the dance group were more likely to be classified at baseline as being at moderate or high risk of falling than those in the control group. Adjustment for this possible confounder did not change our findings, which may suggest that dance programs are not suitable for seniors at high risk of falling. This was confirmed in a subgroup analysis showing that multiple fallers in the intervention group experienced significantly higher rates of falls than multiple fallers in the control group. Systematic reviews have indicated that not all fall prevention group exercise is equally effective for low- and high-risk groups. For example, in a Cochrane review, Tai Chi was found to be less effective for a high-risk group [5]. Several exercise interventions delivered to high-risk groups have also reported excess falls in the intervention group [41,42].

Our study findings could also be due to participants gaining confidence due to the intervention and therefore increasing their exposure to walking and, consequently, experiencing more falls. During the trial, both groups increased their incidental physical activity with the increase, on average, being higher in dance participants than in controls. Cohort studies on the relationship between physical activity levels and falls are inconsistent [43]; in a mixed-sex cohort, increase in hours walked was associated with a higher fall rate in those with initially low levels of physical activity or slow gait speed (<0.8m/s) [44]. Thirty percent of dance participants had such slow gait speeds, compared with only 15% of controls; a differential that may explain the higher fall rate in dance group participants with slow gait speed. By contrast, a U-shaped relationship was found in a cohort of older men only for those without any mobility limitations, once they crossed a threshold of 9,000 steps per day [45]. Comparison between studies is problematic because of the difference in the definition and measurement of the moderators (i.e., mobility limitations, physical activity, etc.), yet it is possible that the greater increase in physical activity in the dance group increased exposure to falls also in the highly active participants without mobility limitations. Additionally, the heterogeneity of the dance groups in terms of physical abilities and cognitive status may have influenced instructors to find the “middle ground” to retain participation, which may have resulted in less challenging classes for those who were more able and more limited in their physical improvements overall.

The apparent significant excess of falls among folk dance participants and the deterioration in postural sway and PPA fall risk, SPPB, and TMT-B score for folk dance “completers” compared to ballroom dancers or control completers is surprising. One plausible explanation relates to the poorer health status of participants in folk dance villages at baseline and throughout the trial (see S1 Table). Yet, due to study design and power limitations, it cannot be determined whether one dance style is more potentially beneficial than the other.

The lack of effect on executive function (i.e., TMT) in our study is in line with findings from other pragmatic RCTs, whether involving dance [46–48] or walking interventions [49,50]. One explanation for this could be the high proportion of active people in our study. Engaging older adults who are generally fit and active may leave little room for improvement if, as a consequence, the brain already exhibits efficient processing. For example, an effect size of 0.4 for TMT difference was found for a walking intervention involving participants who were screened for being “low-active” (average of 4,453 steps per d) [51], but no effect was found in a trial that included older adults who were doing up to 3.5 h/wk of exercise, as in our study [52]. Further, an RCT on the effects of aerobic exercise on cognitive plasticity not only recruited inactive people but also was conducted in laboratory conditions; the study employed a training protocol of high intensity (70% of maximum VO_2_ capacity of each individual) [53], an intensity that is unlikely to be achieved in community social dance programs.

### Strengths and Limitations of the Study

To our knowledge, this is the first RCT providing empirical evidence about the effectiveness of social dancing on the incidence of falls. We used minimal exclusion criteria and achieved a high retention rate (80%). Our sample is representative of the NSW residents who live in retirement villages with regard to sex distribution and living arrangements [54]. Our sample is also representative of the general population of NSW older adults by country of birth, education levels, and fall history [55], but may have been more active than the general older population, and this could have contributed to the results seen. The program was well accepted, and five villages chose to continue social dancing classes after the trial. Further, we showed that the recruitment rate (12%) and retention rate (9.6%) from the entire population of village residents was much higher than the population rate of participation in dance (3.5%) among older Australians [56], which may suggest an unmet demand.

A few limitations must be acknowledged. First, the low attendance to classes precluded reaching a critical “dose” of training for a substantial proportion of participants. Second, the study was conducted as a pragmatic trial; measurements were conducted in the residents’ villages, and therefore the conditions were not uniform across villages in terms of room space, noise, or surface (wood, carpet), and in two villages the baseline and follow-up measurement area were different, which may have impacted on the participants’ performance but is unlikely to have introduced systematic bias. Furthermore, over the trial period, we had seven research assistants conducting the study measurements; hence, there remains some possibility of inter-rater measurement error. A third limitation is the lack of randomisation to dance style and insufficient power for a dance style-based analysis and for detecting smaller effects. It is possible that the selection of two styles and several instructors per style increased the variability in the delivery of the program, which may have affected study outcomes. Fourth, we did not include psychological measures such as risk taking behaviour or participants’ fear of falling to ascertain the supposition that increased confidence or tendency for risk taking may explain the outcome. Finally, we did not include a measure of dynamic balance such as the “narrow walk” test, which may be more sensitive than the postural sway test to any change brought about by dancing.

### Conclusions

This study does not support the inferences from small and short-term RCTs that social dancing programs of different styles can prevent falls or improve fall-risk factors among older adults. More RCTs targeting people separately at high and low risk of falling are needed to establish fall prevention benefits. Adaptation of the program to contain “training elements” to achieve a sufficient balance challenge may be needed. Dancing is classified as a moderate-intensity physical activity, and different dance styles have been shown to have other health benefits such as an increase in cardiorespiratory fitness for patients suffering from chronic heart failure following Waltz dancing [57] and a traditional Greek dance [58], or increased bone density in postmenopausal women at risk of osteoporosis following a line dancing program [18]. It is possible that some dance styles could be more effective for fall prevention than other styles, and this is worth exploring in future RCTs.

## Supporting Information

S1 Table Characteristics of dance participants at baseline and study end by dance style. (DOCX)

S2 Table Number of falls and incidence of falling by dance style and attendance. (DOCX)

S3 Table Baseline and “study-end” scores for the TMT and secondary outcomes by study allocation and completers. (DOCX)

S4 Table Baseline and “study-end” scores for the TMT, and secondary outcomes by dance style among trial completers. (DOCX)

S1 Text CONSORT 2010 checklist when reporting a cluster RCT. (DOCX)

S2 Text TIDieR (Template for Intervention Description and Replication) checklists. (DOCX)

S3 Text Human ethics approval. (DOCX)

S4 Text Published study protocol. (PDF)

## Abbreviations

GEE: Generalized Estimating Equations
IPEQ: Incidental and Planned Exercise Questionnaire
IRR: Incidence Rate Ratio
ITT: intention to treat
MCS: Mental Component Summary
MMSE: Mini Mental State Examination
PCS: Physical Component Summary
GDS: Geriatric Depression Scale
PPA: Physiological Performance Assessment
RCT: randomised controlled trial
SD: standard deviation
SF-12: Short-Form 12
SPPB: Short Physical Performance Battery
TAFE: Technical And Future Education
TMT: Trail Making Test
TMT-B: Trail Making Test Part B
