# Supplementary material for: Social Dancing and Incidence of Falls in Older Adults: A Cluster Randomised Controlled Trial
Source: PLoS Med. 2016 Aug 30;13(8):e1002112. doi: 10.1371/journal.pmed.1002112 (PMC5004860; doi:10.1371/journal.pmed.1002112)
Supplement: S1 Table — (DOCX) [file pmed.1002112.s001.docx]

**S1 Table: Characteristics of dance participants at baseline and study end by dance style**

|  | **Baseli**ne  Folk Dance (N=95) | | | | Ballroom (N=184) | | | | **Study end**  Folk Dance (n=61) | | | | Ballroom (n=150) | | |
| --- | --- | --- | --- | --- | --- | --- | --- | --- | --- | --- | --- | --- | --- | --- | --- |
|  | | n % | | | n % | | | | n % | | n % | | | | |
| **Demographics** | | |  |  | |  |  |  | |  | |  | |  |  |
| Age > 80 years | | | 34 | (36) | | 85 | (46) ^ŧ^ | 23 | | (34) | | 71 | | (47) ^ŧ^ |  |
| Female sex | | | 77 | (81) | | 154 | (84) | 55 | | (82) | | 124 | | (83) |  |
| **Country of Birth** | | |  |  | |  |  |  | |  | |  | |  |  |
| Australia | | | 63 | (66) | | 130 | (71) | 44 | | (66) | | 103 | | (69) |  |
| English-speaking | | | 16 | (17) | | 36 | (19) | 10 | | (15) | | 29 | | (19) |  |
| Non-English speaking | | | 16 | (17) | | 18 | (10) | 13 | | (19) | | 18 | | (12) |  |
| **Highest level of educational attainment** | | |  |  | |  |  |  | |  | |  | |  |  |
| Year 10 or below | | | 27 | (28) | | 61 | (33) | 20 | | (30) | | 49 | | (33) |  |
| Completed High school/ TAFE ^a^ | | | 50 | (53) | | 96 | (52) | 34 | | (51) | | 77 | | (51) |  |
| University degree | | | 18 | (19) | | 27 | (15) | 13 | | (19) | | 24 | | (16) |  |
| **Living alone** | | |  |  | |  |  |  | |  | |  | |  |  |
| Yes | | | 72 | (76) | | 103 | (56)** | 53 | | (79) | | 87 | | (58)** |  |
| **Falls in past year** | | |  |  | |  |  |  | |  | |  | |  |  |
| 0 falls | | | 70 | (74) | | 132 | (72) | 52 | | (77) | | 110 | | (73) |  |
| 1 fall | | | 17 | (18) | | 31 | (17) | 22 | | (16) | | 26 | | (17) |  |
| ≥2 falls (‘multiple fallers’) | | | 7 | (7) | | 20 | (11) | 4 | | (6) | | 14 | | (9) |  |
| **Number of chronic conditions** | | |  |  | |  |  |  | |  | |  | |  |  |
| ≥2 chronic conditions | | | 73 | (78) | | 124 | (67) ^ŧ^ | 52 | | (78) | | 99 | | (66) ^ŧ^ |  |
| **Diseases known to increase falls** | | |  |  | |  |  |  | |  | |  | |  |  |
| Stroke | | | 15 | (16) | | 13 | (3)** | 12 | | (18) | | 11 | | (7)* |  |
| Parkinson’s Disease | | | 2 | (2) | | 1 | (0) | 2 | | (3) | | 0 | | (0)* |  |
| Arthritis | | | 60 | (64) | | 94 | (51)* | 42 | | (63) | | 76 | | (51) ^ŧ^ |  |
| Diabetes | | | 11 | (12) | | 23 | (12) | 9 | | (13) | | 17 | | (11) |  |
| Depression symptoms (GDS >=5) | | | 18 | (19) | | 25 | (14) | 11 | | (16) | | 20 | | (13) |  |
| **Poor vision (**Edge contrast sensitivity < =16)^b^ | | | 3 | ( 3) | | 9 | ( 5) | 3 | | ( 4) | | 7 | | ( 5) |  |
| **Medication use** | | |  |  | |  |  |  | |  | |  | |  |  |
| > 5 medications | | | 42 | (44) | | 75 | (41) | 28 | | (42) | | 60 | | (40) |  |
| Psychoactive medications | | | 7 | (7) | | 10 | (5) | 6 | | (9) | | 6 | | (4) |  |
| **Physiological falls risk profile** | | |  |  | |  |  |  | |  | |  | |  |  |
| No risk  Low  mild  Moderate to High | | | 31  30  22  12 | (33) (32) (23) (12) | | 49  58  42  35 | (27)^ŧ^ (31) (29) (19) | 25  21  14  7 | | (37) (31) (21) (11) | | 40  51  35  24 | | (27) (34) (23) (16) |  |
| **Cognitive status (MMSE)** | | |  |  | |  |  |  | |  | |  | |  |  |
| 28-30 | | | 61 | (64) | | 122 | (66) | 44 | | (66) | | 101 | | (67) |  |
| <27 | | | 34 | (36) | | 62 | (34) | 23 | | (34) | | 49 | | (33) |  |
| **Exercise level** | | |  |  | |  |  |  | |  | |  | |  |  |
| ≥3.0 hrs/week of planned exercise | | | 56 | (60) | | 108 | (59) | 43 | | (65) | | 91 | | (61) |  |
| ≥14.0 hrs/week of physical activity | | | 79 | (85) | | 151 | (82) | 57 | | (86) | | 127 | | (85) |  |
| **Dancing status** | | |  |  | |  |  |  | |  | |  | |  |  |
| Currently dancing | | | 5 | (5) | | 18 | (10) | 5 | | (7) | | 16 | | (11) |  |
| Never danced | | | 22 | (23) | | 50 | (27) | 16 | | (24) | | 40 | | (27) |  |

**p<=0.05 ** p<0.01* ^ŧ^ *marginal p<0.08*
