# Supplementary material for: Social Dancing and Incidence of Falls in Older Adults: A Cluster Randomised Controlled Trial
Source: PLoS Med. 2016 Aug 30;13(8):e1002112. doi: 10.1371/journal.pmed.1002112 (PMC5004860; doi:10.1371/journal.pmed.1002112)
Supplement: S2 Table — (DOCX) [file pmed.1002112.s002.docx]

**S2 Table: Number of falls and incidence of falling by dance style and attendance**

|  | **Intervention** | **Control** | **Unadjusted Adjusted** |
| --- | --- | --- | --- |
|  | **Falls (rate ^a^ )** | **Falls (rate)** | **IRR ^b^ (95% CI) IRR ^c^ (95% CI)** |
| Dance style ^d^ |  |  |  |
| Folk dance (n=95) | 110 (1.34) | 187 (0.80) | 1.74 (1.14-2.65) 1.68 (1.03-2.73) |
| Ballroom (n=182) | 147 (0.88) |  | 1.14 (0.84-1.54) 0.92 (0.65-1.29) |
| Attendance to dancing classes ^d^ |  |  |  |
| Low: <21 classes (n=65) | 95 (2.09) |  | 2.76 (1.65-4.64) 2.34 (1.27-4.31) |
| Medium: 21-44 classes (n=68) | 60 (0.96) | 187 (0.80) | 1.19 (0.66-2.18) 1.02 (0.57-1.84) |
| High: ≥ 45 classes (n=142) | 102 (0.73) |  | 0.91 (0.68-1.23) 0.83 (0.58-1.20) |
| High attendance - folkdance | 46 (0.85) | 187 (0.80) | 1.08 (0.74-1.57) 1.14 (0.75-1.74) |
| High attendance - ballroom | 96 (0.66) |  | 0.84 (0.61-1.16) 0.71 (0.48-1.05) |
|  |  |  |  |
| ^a^=fall rates per person-year ^b^ = IRR (incidence risk ratio) for dance group compared to control group, allowing for cluster ^c^=Adjusted for age, gender, educational attainment, baseline Mini-Mental State Examination, dancing status at baseline, fall risk at baseline . The adjusted model included 521 participants due to 1 person with missing data on dancing status and the adjusted model comparing previous fallers included 141 participants due to 2 participants with no data on history of falls. | | | |
