# Supplementary material for: Social Dancing and Incidence of Falls in Older Adults: A Cluster Randomised Controlled Trial
Source: PLoS Med. 2016 Aug 30;13(8):e1002112. doi: 10.1371/journal.pmed.1002112 (PMC5004860; doi:10.1371/journal.pmed.1002112)
Supplement: S3 Table — (DOCX) [file pmed.1002112.s003.docx]

**S3 Table: Baseline and ‘study-end’ scores for the Trail Making Test and secondary outcomes by study allocation and completers ^a^**

|  | | **Dance (N=275)** | | **Control (N=247)** | | **Intervention effect: adjusted ^c^ between-group difference at 12 months (95% CI)** | |  | | |
| --- | --- | --- | --- | --- | --- | --- | --- | --- | --- | --- |
|  | | **Baseline**  **Mean (SD)** | **12 months**^b^  **Mean (SD)** | **Baseline**  **Mean (SD)** | **12 months^b^**  **Mean (SD)** |  |  | ***P-value* ^c^** | | |
| TMT ^d^ A (s) (n=422) | | 43.5 (16.6) | 47.0 (22.0) | 40.0 (13.7) | 42.2 (17.9) | 0.6 | (-2.2, 3.4) | | 0.67 | |
| TMT ^d^ B (s) | | 119.0 (59.6) | 129.0 (73.8) | 118.4 (64.0) | 120.0 (63.3) | 0.7 | (0.6, 0.8) | | 0.34 | |
| TMT ^d^ difference (s) | | 75.5 (49.8) | 82.0 (64.4) | 78.4 (56.3) | 77.7 (53.9) | 1.7 | (-6.7, 10.1) | | 0.69 | |
| PPA^e^ total score (n=419) | | 0.69 (1.17) | 0.85 (1.30) | 0.46 (1.04) | 0.52 (1.07) | 0.20 | (-0.15, 0.56) | | 0.26 | |
| Proprioception (degrees) | | 1.86 (1.22) | 2.07 (1.37) | 1.85 (1.13) | 2.01 (1.20) | 0.08 | (-0.22, 0.38) | | 0.60 | |
| Leg strength (kg) | | 22.4 (12.7) | 26.0 (11.9) | 23.1 (10.8) | 26.6 (12.2) | 0.5 | (-4.6, 5.5) | | 0.86 | |
| Postural sway path (mm) | | 165 (131) | 180 (197) | 137 (106) | 124 (84) | 1.31 | (1.03, 1.65) ^h^ | | 0.03 | |
| Reaction time ( s) | | 253 (59) | 265 (82) | 251 (55) | 262 (67) | 1.00 | (0.95, 1.06) ^h^ | | 0.98 | |
| SPPB ^f^ Score (n=410) | | 10.3 (1.7) | 10.1 (2.5) | 10.7 (1.7) | 10.5 (2.1) | -0.3 | (-0.7, 0.2) | | 0.28 | |
| 5-repeats STS (s, n=393) | | 12.0 (3.4) | 12.1 (4.2) | 11.8 (3.6) | 11.9 (4.4) | 0.0 | (-0.6, 0.7) | | 0.95 | |
| Gait speed (m/s, n=412) | | 0.97 (0.25) | 1.00 (0.24) | 1.02 (0.22) | 0.99 (0.20) | 0.05 | (0.02, 0.12) | | 0.13 | |
| Quality of Life ^g^ (n=414) | |  |  |  |  |  | | |  | |
| Physical component score | | 43.2 (8.6) | 41.8 (10.3) | 44.6 (8.7) | 42.6 (9.9) | 0.5 | (-1.0, 2.0) | | 0.45 | |
| Mental component score | | 53.0 (8.1) | 52.3 (8.7) | 51.9 (7.4) | 51.8 (8.2) | -0.2 | (-1.5, 1.2) | | 0.82 | |
|  | ^a^ =completers refer to participants who attended the ‘study-end’ measurement session and had complete data at both time measurement ^b^= Missing data were estimated by multiple imputation using chained equations (with 30 imputed datasets ^c^= Between groups difference in 12 months scores for all outcomes were assessed using Generalized Estimating Equations (GEE) adjusted for baseline outcome values and age, gender, educational attainment, Mini-Mental State Examination Score and dancing status, and accounting for retirement village clustering effect.  ^d^ =Trail Making Test ^e^ =Physiological Performance Assessment ^f^ =Short Physical Performance Battery ^g^ = Short Form 12 survey ^h^ = represents antilogarithm of regression coefficient as GEE using logarithmic link function; value represents proportional difference in outcome between the Dance and Control groups. | | | | | | | | |  |
