# Supplementary material for: Social Dancing and Incidence of Falls in Older Adults: A Cluster Randomised Controlled Trial
Source: PLoS Med. 2016 Aug 30;13(8):e1002112. doi: 10.1371/journal.pmed.1002112 (PMC5004860; doi:10.1371/journal.pmed.1002112)
Supplement: S4 Table — (DOCX) [file pmed.1002112.s004.docx]

**S4 Table: Baseline and ‘study-end’ scores for the Trail Making Test and secondary outcomes by dance style among trial completers ^a^**

|  | **Folk dance (n=67)** | | **Ballroom dance (n=150)** | | **Control (n=207)** | | **Folk vs. Control** | | **Ballroom vs. Control** | |
| --- | --- | --- | --- | --- | --- | --- | --- | --- | --- | --- |
| Outcome | **Baseline**  **Mean**  **(SD)** | **12 months**  **Mean**  **(SD)** | **Baseline**  **Mean**  **(SD)** | **12**  **months**  **Mean**  **(SD)** | **Baseline Mean**  **(SD)** | **12**  **months**  **Mean**  **(SD)** | **Interven-tion effect ^b^**  **(95% CI)** | ***P* ^b^** | **Interven- tion effect ^b^**  **(95% CI)** | ***P* ^b^** |
| TMT ^c^ A (s) (n=421) | 43.5  (17.0) | 48.0  (22.3) | 43.6  (16.4) | 46.5  (22.0) | 40.0  (13.7) | 42.2  (17.9) | 1.9  (-2.3, 5.9) | 0.377 | 0.1  (-3.3, 3.4) | 0.97 |
| TMT ^c^ B (s) (n=421) | 124.7  (66.0) | 138.6  (84.0) | 116.4  (56.5) | 124.8  (68.7) | 118.4  (64.0) | 120.0  (63.3) | 10.7  (3.5, 17.9) | 0.004 | 0.4  (-7.9, 8.8) | 0.92 |
| TMT ^c^ difference (s) (n=421) | 81.1  (57.5) | 90.6  (75.2) | 73.8  (47.0) | 78.3  (58.6) | 78.4  (56.3) | 77.7  (53.9) | 8.9  (-1.5, 19.2) | 0.092 | -2.6  (-12.0, 6.9) | 0.59 |
| PPA^d^ total score (n=418) | 0.44  (1.15) | 0.87  (1.44) | 0.80  (1.16) | 0.85  (1.24) | 0.46  (1.04) | 0.52  (1.07) | 0.17  (-0.11, 0.45) | 0.231 | 0.14  (-0.26, 0.54) | 0.48 |
| Proprioception (degrees) | 1.75  (1.52) | 2.31  (1.71) | 1.89  (1.06) | 1.97  (1.18) | 1.85  (1.13) | 2.01  (1.20) | 0.41  (-0.15, 0.97) | 0.150 | -0.07  (-0.34, 0.20) | 0.62 |
| Leg strength (kg) | 19.1  (12.1) | 23.3  (13.1) | 23.6  (12.7) | 27.2  (11.3) | 23.1  (10.8) | 26.6  (12.2) | -0.7  (-5.1, 3.7) | 0.750 | 1.1  (-5.0, 7.1) | 0.73 |
| Postural sway path (mm) | 133  (88) | 171  (202) | 176  (139) | 185  (194) | 137  (106) | 124  (84) | 1.31 ^g^  (0.96, 1.77) | 0.089 | 1.29 ^g^  (0.95, 1.74) | 0.10 |
| Reaction time ( s) | 249  (72) | 278  (112) | 254  (52) | 260  (64) | 251  (55) | 262  (67) | 1.06 ^g^  (0.99, 1.13) | 0.114 | 0.97 ^g^  (0.94, 1.01) | 0.14 |
| SPPB ^e^ Score (n=419) | 10.3  (1.6) | 9.5  (3.3) | 10.3  (1.7) | 10.4  (2.0) | 10.7  (1.7) | 10.5  (2.1) | -0.9  (-1.4, -0.4) | 0.001 | 0.1  (-0.4, 0.5) | 0.85 |
| 5-repeats STS (s, n=392) | 11.3  (3.0) | 12.1  (3.9) | 12.2  (3.6) | 12.2  (4.4) | 11.8  (3.6) | 11.9  (4.4) | 0.7  (0.1, 1.3) | 0.034 | -0.3  (-0.8, 0.3) | 0.38 |
| Gait speed (m/s, n=412) | 0.99  (0.28) | 0.96  (0.22) | 0.96  (0.23) | 1.02  (0.25) | 1.02  (0.22) | 0.99  (0.20) | -0.01  (-0.08, 0.06) | 0.757 | 0.07  (0.00, 0.14) | 0.05 |
| Quality of Life ^f^ (n = 414) |  |  |  |  |  |  |  |  |  |  |
| Physical component score | 42.6  (9.0) | 40.6  (12.0) | 43.4  (8.4) | 42.3  (9.4) | 44.6  (8.7) | 42.6  (9.4) | -0.6  (-2.7, 1.8) | 0.581 | 1.0  (-0.6, 2.6) | 0.23 |
| Mental component score | 53.3  (7.4) | 51.2  (10.7) | 52.9  (8.4) | 52.7  (7.6) | 51.9  (7.4) | 51.8  (8.2) | -1.5  (-3.7, 0.7) | 0.187 | 0.5  (-0.9, 1.9) | 0.51 |
| ^a^ =completers refer to participants who attended the ‘study-end’ measurement n=424, analysis was limited to available data. ^b^= Between groups difference in 12 months scores was tested when each style was compared to control. All outcomes were assessed using Generalized Estimating Equations (GEE) adjusted for age, gender, educational attainment, and baseline outcome values, Mini-Mental State Examination Score and dancing status, and accounting for retirement village clustering effect. clustering effect ^c^ =Trail Making Test ^d^ =Physiological Performance Assessment ^e^ =Short Physical Performance Battery  ^f^ = Short Form 12 (SF-12) survey ^g^ = represents antilogarithm of regression coefficient as GEE using logarithmic link function; value represents proportional difference in outcome between the Dance and Control groups. | | | | | | | | | | |
