# Supplementary material for: Social Dancing and Incidence of Falls in Older Adults: A Cluster Randomised Controlled Trial
Source: PLoS Med. 2016 Aug 30;13(8):e1002112. doi: 10.1371/journal.pmed.1002112 (PMC5004860; doi:10.1371/journal.pmed.1002112)
Supplement: S2 Text — (DOCX) [file pmed.1002112.s006.docx]

**
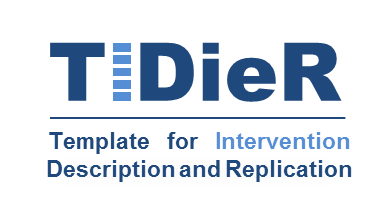
The TIDieR (Template for Intervention Description and Replication) Checklist*:**

Information to include when describing an intervention and the location of the information

| **Item number** | **Item** | **Where located **** | |
| --- | --- | --- | --- |
|  |  | Primary paper  (page or appendix  number) | Other ^†^ (details) |
|  | **BRIEF NAME** |  |  |
| **1.** | Provide the name or a phrase that describes the intervention. | Social dancing Folkdance & ballroom | Abstract 2^nd^ paragraph; Methods subheading Intervention and Control |
|  | **WHY** |  |  |
| **2.** | Describe any rationale, theory, or goal of the elements essential to the intervention. | Small-scale RCT showed a variety of dance styles can improve functional balance and gait- two major risk factors for falls. However, some RCT included warm-up and sections of specific balance training. We aimed to test social dancing as delivered in the community. | Background-3^rd^ paragraph and last paragraph. |
|  | **WHAT** |  |  |
| **3.** | Materials: Describe any physical or informational materials used in the intervention, including those provided to participants or used in intervention delivery or in training of intervention providers. Provide information on where the materials can be accessed (e.g. online appendix, URL). | Ballroom- Rock &Roll, Foxtrot, Waltz, Salsa and Rumba. Folkdance- from UK, US, France, Italy, Israel and Greece | We can provide DVD and teachers booklet if needed |
| **4.** | Procedures: Describe each of the procedures, activities, and/or processes used in the intervention, including any enabling or support activities. | Folkdance group were asked to use the Borg Rate of Perceived Exertion in classes 20,40, 60 and 80. | Methods subheading Participants’ involvement. |
|  | **WHO PROVIDED** |  |  |
| **5.** | For each category of intervention provider (e.g. psychologist, nursing assistant), describe their expertise, background and any specific training given. | Eight experiences dance instructors (4 from each style) delivered the programs. They were given two workshops (before start) and at 6 months to standerdized the program, and booklet to follow. | Methods subheading “Intervention- and in the subheading Participants involvement” , which provides the process of teachers’ selection using demonstration classes |
|  | **HOW** |  |  |
| **6.** | Describe the modes of delivery (e.g. face-to-face or by some other mechanism, such as internet or telephone) of the intervention and whether it was provided individually or in a group. | Face to face | Methods subheading “intervention” |
|  | **WHERE** |  |  |
| **7.** | Describe the type(s) of location(s) where the intervention occurred, including any necessary infrastructure or relevant features. | In retirement villages community halls or other room enough to accommodate the group | Eligibility criteria for site – Methods subheading Recruitment and particioants. |
|  | **WHEN and HOW MUCH** |  |  |
| **8.** | Describe the number of times the intervention was delivered and over what period of time including the number of sessions, their schedule, and their duration, intensity or dose. | Twice weekly for 1 hour along 12 months but overall 80 classes | Methods subheading “Intervention” – |
|  | **TAILORING** |  |  |
| **9.** | If the intervention was planned to be personalised, titrated or adapted, then describe what, why, when, and how. | No tailoring to high-low risk participants or to experienced and not experienced dancers | Discussion-subheading “Main results in the context of other research” 1^st^ paragraph emphasise the difference between our intervention to other pilot studies. In the Conclusion: tailoring was suggested for future studies |
|  | **MODIFICATIONS** |  |  |
| **10.^ǂ^** | If the intervention was modified during the course of the study, describe the changes (what, why, when, and how). | No changes to the program was made - but in several villages the progression was slower than expected | Discussion –subheading  “Main results in the context of other research” at the end of the 4^th^ paragraph. |
|  | **HOW WELL** |  |  |
| **11.** | Planned: If intervention adherence or fidelity was assessed, describe how and by whom, and if any strategies were used to maintain or improve fidelity, describe them. | Each teacher received a Diary to take attendance and write comments. They were instructed to report the field coordinator on any harm during the trial (such as a fall) and to contact participants who did not showed to class more than 2 weeks to verify continuation. | Methods –subheading Intervention- |
| **12.^ǂ^** | Actual: If intervention adherence or fidelity was assessed, describe the extent to which the intervention was delivered as planned. | Mostly delivered as planned; ballroom complicated steps were not taught in slow classes. | Discussion- subheading “Main results in the context of other research” 4^th^ paragraph. |

** **Authors** - use N/A if an item is not applicable for the intervention being described. **Reviewers** – use ‘?’ if information about the element is not reported/not sufficiently reported.

† If the information is not provided in the primary paper, give details of where this information is available. This may include locations such as a published protocol or other published papers (provide citation details) or a website (provide the URL).

ǂ If completing the TIDieR checklist for a protocol, these items are not relevant to the protocol and cannot be described until the study is complete.

* We strongly recommend using this checklist in conjunction with the TIDieR guide (see *BMJ* 2014;348:g1687) which contains an explanation and elaboration for each item.

* The focus of TIDieR is on reporting details of the intervention elements (and where relevant, comparison elements) of a study. Other elements and methodological features of studies are covered by other reporting statements and checklists and have not been duplicated as part of the TIDieR checklist. When a **randomised trial** is being reported, the TIDieR checklist should be used in conjunction with the CONSORT statement (see [www.consort-statement.org](http://www.consort-statement.org)) as an extension of **Item 5 of the CONSORT 2010 Statement.** When a **clinical trial** **protocol** is being reported, the TIDieR checklist should be used in conjunction with the SPIRIT statement as an extension of **Item 11 of the SPIRIT 2013 Statement** (see [www.spirit-statement.org](http://www.spirit-statement.org)). For alternate study designs, TIDieR can be used in conjunction with the appropriate checklist for that study design (see [www.equator-network.org](http://www.equator-network.org)).
