# Supplementary material for: Social Dancing and Incidence of Falls in Older Adults: A Cluster Randomised Controlled Trial
Source: PLoS Med. 2016 Aug 30;13(8):e1002112. doi: 10.1371/journal.pmed.1002112 (PMC5004860; doi:10.1371/journal.pmed.1002112)
Supplement: S3 Text — (DOCX) [file pmed.1002112.s007.docx]

# **UWS HUMAN RESEARCH ETHICS COMMITTEE**

19 March 2012

Associate Professor Dafna Merom,

School of Science and Health

Dear Dafna,

I wish to formally advise you that the Human Research Ethics Committee has approved your research proposal **H9468** *“The effectiveness of social dancing as a strategy to prevent falls in order people“,* until 20 March 2015 with the provision of a progress report annually and a final report on completion.

Please quote the project number and title as indicated above on all correspondence related to this project.

This protocol covers the following researchers:

Dafna Merom, Cathie Sherrington, Margaret Connor, Kaarin Anstey, Robert Cumming, Peter van Vilet, Ian Hunter, Ester Cerin, Chris Rissel, Stephen Lord.

Yours sincerely

Dr Anne Abraham

Chair, UWS Human Research Ethics Committee
