# Supplementary material for: Social Dancing and Incidence of Falls in Older Adults: A Cluster Randomised Controlled Trial
Source: PLoS Med. 2016 Aug 30;13(8):e1002112. doi: 10.1371/journal.pmed.1002112 (PMC5004860; doi:10.1371/journal.pmed.1002112)
Supplement: S4 Text — (PDF) [file pmed.1002112.s008.pdf]

Please note: All data submitted to this register will be made publicly available.

ANZCTR will be unattended over the festive season from Wednesday, 19th December 2012 and re-opening on Wednesday, 2nd January 2013.

Questions in **bold** text are mandatory. **(*)**

**Request Number:**	362450
**Current Page:**	Review

View Trial at Registration | View History

Trial from ANZCTR

**Trial ID**	ACTRN12612000889853
**Trial Status:**	Registered
**Date Submitted:**	13/08/2012
**Date Registered:**	21/08/2012

Page 1

Edit

**Public title**	The effectiveness of social dancing as a strategy to prevent falls in older people
**Study title in 'Participant-Intervention-Comparator- Outcome (PICO)' format**	Evaluation of social dancing compared to wait list controls in reducing incidence of falls in older people
**Secondary ID [1]**	Nil
UTN	U1111-1130-3798
Trial acronym	DAnCE (Dance, AgeiNg, Cognition and Economics) fall prevention trial

Page 2

Edit

Health condition(s) or problem(s) studied:

Prevention of falls in older adults

Prevention of cognitive decline in older adults

**Condition category:**	**Condition code:**

Musculoskeletal	Other muscular and skeletal disorders
Mental Health	Studies of normal psychology, cognitive function and behaviour
Public Health	Health promotion/education

Page 3

Edit

**Descriptions of intervention(s) / exposure**	Social Dancing (folk and ballroom dancing) in groups delivered by certified dance instructors with particular experience in teaching older adults. The classes are 1-hour long delivered twice weekly, in total 80 hours over 12 months.
**Intervention Code:**	Prevention
**Intervention Code:**	Lifestyle
**Comparator / control treatment**	Participants in the control group will be advised to continue with their regular activities. They will receive monthly newsletters containing study updates, “meet the team” section and health education section. The topics for the educational sections are: Vitamin D, sleeping habits, Healthy eating, Smoking cessation, Appropriate footwear, Reducing alcohol intake, Falls statistics, Aging and the mind, Keeping the mind sharp, Sitting for too long, it is never too old to start, and the Origin of ballroom/folk dance.<br>Controls will be placed on a wait list for the dance classes. Controls will receive 80 hours of dance classes at the completion of the trial.
**Control group**	Active

Page 4

Edit

**Primary Outcome:**	Number of accidental falls per participant. Falls will be recorded monthly for 12 months on a diary (calendar) and be collected by mail. Participants will need to record 'F' (i.e., if a Fall) or 'N' (no fall) every day of the month. Every reported fall will be further contacted by phone to complete details about that fall (where was it, did it ended with injury, type of injury and treatment if applicable). Participants with missing diaries will be contacted by phone, fill the diary with the researcher and will be asked also to mail the diary. In addition, the monthly diary will collect information on utilization of health services for economic evaluation using a list of codes that is printed on the calendar; a visit to GP, any use of a specialist (i.e., physiotherapists, consultation with medical specialist) , visit to an outpatient Clinic, Emergency room, use of ambulance and whether there was a change in medications reported at baseline.
***Timepoint**:*	12 months
**Primary Outcome:**	Cognitive function - Trail Making Test A and B Time to complete the Trail Making Test (TMT, part A and B) will be assessed. The TMT A and B is an executive functioning tests. Part A measures processing speed and involves participants connecting consecutive numbers (e.g., 1-2-3), and Part B is a measure of executive function of ?task shifting? and involves participants connecting alternating letters and numbers (e.g.,1-A-2-B). The difference in time between the two parts will be calculated to isolate the executive component of this test.
***Timepoint**:*	12 months
**Secondary Outcome:**	Physiological Profile Assessment (PPA). The PPA comprises of five tests which provide an overall falls risk score: Vision (edge contrast sensitivity), peripheral sensation (proprioception), lower extremity strength (knee extension), simple reaction time using figure press as the response, and body sway when standing on the medium-density foam rubber mat, which is a measure of balance.
Timepoint:	12 months

**Secondary Outcome:**	Choice Stepping Reaction Time Tests without and with cognitive load. Three short tests which requires the subject to perform quick, correctly targeted steps in response to visual cues. The tests comprises of arrows appear randomly on a computer screen. The arrows have graphical presentations on a dance MAT (front left, front right, side left, side right). Test 1: Participants are asked to step as quickly as possible onto the corresponding arrow of the mat and returned to the centre. The time to react and the time it takes to return to the centre is recorded using a customised software. This test measures The tests measure visual attention, central processing, reaction time and movement time. Test 2: Similar test are then performed but with a no-go task which determine by the colour of the errors (i.e., blue color to inhibit response). The time it takes to respond and step back plus the number of errors are recorded. This test also measures as above plus response inhibition. Test 3: this test is the modification of the Stroop Color-Word to a motion. Participants are shown "congruent arrow" (i.e., the direction of the arrow and the word "front" are similar) and incongruent step by the word. Time and errors are recorded. This test measures response inhibition and selective attention which is an executive function measure.
Timepoint:	12 months
**Secondary Outcome:**	Colour Choice Reaction Time Test - this test requires minimal locomotion but measures reaction time with response inhibition. The test involves the presentation of targets of different colours in one of four cells in a square grid. Participants respond to targets by using hand buttons or foot pedals. Blue target requires a response inhibition (i.e., not pressing any buttons). The computer recorded reaction times and errors (accuracy).
Timepoint:	12 Months
**Secondary Outcome:**	Rey Auditory Verbal Learning test (RAVLT). The test evaluates verbal learning and memory, including proactive inhibition, retroactive inhibition, retention, encoding versus retrieval, and subjective organisation. The test starts with a list of 15 words, which an examiner reads aloud at the rate of one per second. The participant's task is to repeat all the words he or she can remember, in any order. This procedure is carried out a total of five times. Then the examiner presents a second list of 15 words, allowing the participant only one attempt at recall. Immediately following this, the patient is asked to remember as many words as possible from the first list.
Timepoint:	12 months
**Secondary Outcome:**	The Short Physical Performance Battery (SPPB) measures lower limb functional status by assessing balance, gait, strength and endurance of the lower extremities. The tests include side-by-side, semi-tandem, and tandem standing tests to complete 10 seconds, time to complete 3-meter walk at normal speed , and a timed sit-to-stand 5 repeat.
Timepoint:	12 months
**Secondary Outcome:**	Self-reported Quality of Life questionnaires- SF12/SF6
Timepoint:	3 months and 12 months
**Secondary Outcome:**	Self-reported Quality of life questionnaire - ICECAP-O
Timepoint:	3 months and 12 months
**Secondary Outcome:**	$ per fall prevented. Costs for total health care resource utilisation, rather than only falls-related healthcare utilisation will be collected monthly for 12 months. Participants will report on the falls diaries any inpatient hospital admissions, Emergency department visits, Ambulance trips, Doctor and allied health visits, prescribed medications, Home care / nursing care,Nursing home or long term care admission. The cost for each of these item will be extrapolated from the relevant source (e.g., Medicare Benefits Scheme fee for service, PBS for medications, etc..). Participant out-of-pocket costs and care provided by family and /or friends will not be included. The cost of the trial is itemized in the trial budget.
Timepoint:	12 months

Page 5

Edit

**Key inclusion criteria**	1) self care retirement villages with 60 or more residents.<br>2) resident of a selected self care village
**Minimum age**	55 Years
**Maximum age**	0 No limit
**Gender**	Both males and females
**Healthy volunteers?**	Yes
**Key exclusion criteria**	1) Unstable medical condition involving the neuromuscular, skeletal or cardiovascular system<br>2) Mini-mental state examination score <24 indicating cognitive impairment

Page 6

Edit

**Study type**	Interventional
**Purpose of the study**	Prevention
**Allocation to intervention**	Randomised controlled trial
Describe the procedure for enrolling a subject and allocating the treatment (allocation concealment procedures)	All 126 retirement villages in Sydney Greater Metropolitan Area will be contacted by letter to determine eligibility and interest in participating. The first 28 eligible and interested villages to respond will be invited to participate in the trial. Invitations will be sent to all residents of the participating villages, and an information session will be held in order to recruit individual participants. Researchers will attend the retirement village and conduct a telephone screening with potential participants. Once eligibility is established, baseline measures will be collected. Once recruitment and baseline data collection at each village is complete, that village will be randomised. Randomisation will be by minimisation of two of the baseline variables (physiological performance assessment “FallScreen” and Trail Making Part B) which are associated with the primary outcome. The randomisation will be performed by the study statistician using a computer program that allocates the village with probability 0.8 to the group that minimises the imbalance of these variables between intervention and control groups. The study co-ordinator will telephone the study statistician and be advised of the village’s allocation, and will arrange for the delivery of the intervention if appropriate. Allocation will thus be concealed from the research team recruiting villages and participants, as randomisation will occur after baseline measures have been completed.
Describe the methods used to generate the sequence in which subjects will be randomised (sequence generation)	Computer generated randomisation using minimisation
Masking / blinding	Open (masking not used)
Who is / are masked / blinded (choose all that apply)	
Assignment	Parallel
Other design features	Cluster randomised trial
Type of endpoint(s)	Efficacy
Statistical Methods/Analysis	

Page 7

Edit

Phase	Not Applicable
**Anticipated date of first participant enrolment**	22/08/2012
Date of first participant enrolment	
Anticipated date last patient recruited/enrolled	
Actual date last patient recruited/enrolled	
**Target sample size**	460
**Recruitment status (at time of registration)**	Not yet recruiting

Recruitment in Australia

**Recruitment state(s)**	

Page 8

Edit

**Funding Source:**	Government funding body
**Name:**	NHMRC
**Address:**	NHMRC
**Country:**	Australia
**Primary Sponsor**	University
**Name:**	University of Western Sydney
**Address:**	School of Science and Health Building 24 Level 4 Rm.30<br>Campbelltown campus<br>University of Western Sydney<br>Locked bag 1797 Penrith NSW 2751, Australia
**Country:**	Australia
**Secondary Sponsor:**	University
**Name:**	University of Sydney
**Address:**	School of Public Health Edward Ford Building (A27) University of Sydney, 2006
**Country:**	Australia

Page 9

Edit

**Has the study received approval from at least one**	Yes

**Ethics Committee?**	
Ethics Committee name:	University of Western Sydney
Address:	University of Western Sydney Locked bag 1797 Penrith NSW 2751, Australia
Country:	Australia
Approval Date:	
Submitted Date:	
HREC:	H9468
Brief summary	The study will determine whether participating in 12 months social dancing can reduce the incidence of falls and improve physiological performance and cognitive tasks that are associated with greater risk of falling. A cluster randomised controlled trial of 28 self care retirement villages is proposed. Villages will be eligible to take part in the study if they: have at least 60 independent residents; have a common facility area for dancing; do not currently offer dance classes; and provide written consent to the study. Agreeing villages will be randomised 1:1 to intervention or control group. Participants in the 14 control sites will receive no intervention for 12 months, except mailing of educational materials on healthy ageing. They will be offered the same dance program free of charge at the end of the study. In the intervention sites a twice-weekly one-hour ballroom dancing (7 sites) or folk dancing (7 sites) class will be offered. Dance instructors will receive workshops to standardise program delivery across sites. Recruitment will be staggered over a 12 month period. Eligible participants will undergo a 1 hr baseline assessment session, which will comprise a structured interview, psychological and physiological tests. The same measurements will be repeated at 12 month follow-up. A falls diary will be collected each month by mail from all participants. Participants with missing diaries will be contacted by phone. To monitor changes in activity levels, participants will be asked to complete a self-report physical activity questionnaire at 6 months. To assess the intensity level of the dance class, participants will be asked to wear accelerometers periodically. The cost effectiveness of this intervention will also be evaluated at 3 months and 12 months.
Trial website	
Trial related presentations / publications	
Public Notes	

Page 10

Edit

Principal Investigator

**Title:**	
**Name:**	
**Address:**	
**Country:**	
**Tel:**	
Fax:	
**Email:**	

Contact person for public queries

**Name:**	Associate Professor Dafna Merom

**Address:**	University of Western Sydney Locked bag 1797 Penrith NSW 2751, Australia
**Country:**	Australia
**Tel:**	+61 2 46203796
Fax:	
**Email:**	d.merom@uws.edu.au

Contact person for scientific queries

**Title:**	
**Name:**	Associate Professor Dafna Merom
**Address:**	University of Western Sydney Locked bag 1797 Penrith NSW 2751, Australia
**Country:**	Australia
**Tel:**	+61-2-46203796
Fax:	
**Email:**	d.merom@uws.edu.au

Contact person responsible for updating information

Title:	
Name:	Erin Mathieu
Address:	University of Western Sydney Locked bag 1797 Penrith NSW 2751, Australia
Country:	Australia
Tel:	61 2 4620 3895
Fax:	
Email:	e.mathieu@uws.edu.au

Private Notes

Previous Submit

© 2007 ANZCTR :: Terms and Conditions :: Privacy Design by Symbiation
